# Symbiotic microorganisms in *Puto superbus* (Leonardi, 1907) (Insecta, Hemiptera, Coccomorpha: Putoidae)

**DOI:** 10.1007/s00709-017-1135-7

**Published:** 2017-06-30

**Authors:** Teresa Szklarzewicz, Małgorzata Kalandyk-Kołodziejczyk, Katarzyna Michalik, Władysława Jankowska, Anna Michalik

**Affiliations:** 1 Department of Developmental Biology and Morphology of Invertebrates, Institute of Zoology and Biomedical Research, Jagiellonian University, Gronostajowa 9, 30-387 Kraków, Poland; 2 Department of Zoology, Faculty of Biology and Environmental Protection, University of Silesia, Bankowa 9, 40-007 Katowice, Poland

**Keywords:** Symbiotic microorganisms, *Sodalis*-like symbionts, *Wolbachia*, Bacteriocytes, Scale insects

## Abstract

The scale insect *Puto superbus* (Putoidae) lives in mutualistic symbiotic association with bacteria. Molecular phylogenetic analyses have revealed that symbionts of *P. superbus* belong to the gammaproteobacterial genus *Sodalis.* In the adult females, symbionts occur both in the bacteriocytes constituting compact bacteriomes and in individual bacteriocytes, which are dispersed among ovarioles. The bacteriocytes also house a few small, rod-shaped *Wolbachia* bacteria in addition to the numerous large, elongated *Sodalis*-allied bacteria. The symbiotic microorganisms are transovarially transmitted from generation to generation. In adult females which have choriogenic oocytes in the ovarioles, the bacteriocytes gather around the basal part of the tropharium. Next, the entire bacteriocytes pass through the follicular epithelium surrounding the neck region of the ovariole and enter the space between oocyte and follicular epithelium (perivitelline space). In the perivitelline space, the bacteriocytes assemble extracellularly in the deep depression of the oolemma at the anterior pole of the oocyte, forming a “symbiont ball”.

## Introduction

Scale insects (coccoids) constitute the superfamily Coccoidea assigned to the infraorder Coccomorpha, within the suborder Sternorrhyncha of the order Hemiptera (Williams and Hodgson 2014). This superfamily is composed of about 8000 species. Scale insects are plant sap-feeding insects, which are distributed in all terrestrial zoogeographical regions, except Antarctica. Many species are thought to be serious pests of economic significance (Kondo et al. 2008; Gullan and Martin 2009; Gertsson 2013). Scale insects are much more diverse in terms of their internal and external morphology, chromosome systems, sperm structure, and types of bacterial symbioses than any of the other sternorrhynchan groups (reviewed in Miller and Kosztarab 1979; Gullan and Kosztarab 1997). The Coccoidea is more frequently divided into two informal groups, which are sometimes treated as superfamilies: the archaeococcoids (Orthezioidea) and the neococcoids (Coccoidea sensu stricto) (e.g., Koteja 1974, 1996; Danzig 1980; Kosztarab and Kozár 1988).

The evolutionary relationships, higher classification, and the taxonomic placement of many species of coccoids are controversial (Cook et al. 2002; Gullan and Cook 2007). One of the species of uncertain taxonomic position is *Puto superbus* (Leonardi 1907). *Puto superbus* was described as *Macrocerococcus superbus* by Leonardi (1907). The name *Macrocerococcus superbus* was used by several authors (e.g., Borchsenius 1949; Schmutterer 1952), although other authors assigned this species to different genera: i.e., *Ceroputo* Sulc and *Phenacoccus* Cockerell (García Morales et al. 2016)***.*** The name *Puto superbus* has been widely accepted and is currently most commonly used (e.g., Marotta and Tranfaglia 1985; Kaydan et al. 2015).

For many years, *Puto superbus* was considered to be a member of the family Pseudococcidae (mealybugs) (e.g., Leonardi 1907; Kosztarab and Kozár 1988). Beardsley (1969) was the first to place *Puto* in the separate Putoidae family based on male features. The Putoidae family has been placed among neococcoids (Koteja 1996; Koteja and Azar 2008) or archaeococcoids (Gullan and Cook 2007; Kondo et al. 2008; Gullan and Martin 2009). The placement of this family within archaeococcoid subgroup was supported by molecular and ultrastructural data (Cook et al. 2002; Gullan and Cook 2007; Michalik et al. 2013). The Putoidae contains two genera: *Palaeoputo* Koteja and Azar 2008 which encompasses only one fossil species and *Puto* Signoret, 1876 with 2 extinct and 45 extant species (García Morales et al. 2016)***.***

Scale insects, like other plant sap-feeders, are host to obligate symbiotic microorganisms, which synthesize and provide them the amino acids missing from their diet (reviewed, e.g., in Douglas 1998, 2009; Wilkinson and Ishikawa 2001; Ishikawa 2003; Baumann 2005, 2006).

In insects living in symbiotic association with two or more species of symbiotic microorganisms, Buchner (1965) divided the latter into primary symbionts (named also P-symbionts or obligate symbionts) and accessory symbionts (also known as S-symbionts, facultative symbionts, and secondary symbionts). Since obligate symbionts are the descendants of a free-living microorganism that infected the ancestor of the insect taxon, they occur in all the individuals of each species of this group of insects (e.g., bacterium *Buchnera aphidicola* in aphids). As these microorganisms are responsible for the synthesis of amino acids missing in their diet, they are necessary for the proper growth and reproduction of the host insect (Moran and Dale 2006; Moran et al. 2008). On the other hand, the occurrence of S-symbionts in insects is a result of more recent multiple independent infections by different microorganisms (Moran and Dale 2006; Moran and Telang 1998). As a consequence, the S-symbionts living even in close relatives may belong to different taxa. The results of experiments on a model aphid, *Acyrthosiphon pisum*, indicate that in contrast to P-symbionts, S-symbionts may play different roles, e.g., protecting the host insect against parasitic hymenopterans or fungal pathogens or increase heat tolerance (Montllor et al. 2002; Oliver et al. 2003; Scarborough et al. 2005). In most insects, obligate symbionts are harbored in specialized cells of a mesodermal origin, termed bacteriocytes (the older term being “mycetocytes”), and are always vertically (maternally) transmitted from one generation to the next. S-symbionts may occur both intracellularly (e.g., in bacteriocytes, in cells of midgut epithelium) and extracellularly (e.g., in hemolymph), and may be transferred both vertically and horizontally (i.e., between individuals of the same population). It should be added that Takiya et al. (2006) on account of occurrence in Hemiptera: Auchenorrhyncha of several types of symbionts involved in the synthesis of amino acids to host insects distinguished the third type of symbionts and termed them ‘co-primary’ symbionts.

The results of earlier histological studies and more recent ultrastructural and molecular analyses have shown that scale insects, in comparison with other hemipterans, are characterized by diverse symbiotic systems in terms of localization of symbionts in the host insect body, their systematic affinity, and mode of inheritance from the mother to offspring. Scale insects may harbor only one species of symbiotic microorganism (e.g., *Acanthococcus aceris* and *Gossyparia spuria* (both Eriococcidae) (Michalik et al. 2016), members of the mealybug subfamily Phenacoccinae (Gruwell et al. 2010; Koga et al. 2013), most armored scale insects, Diaspididae) (Gruwell et al. 2012; Sabree et al. 2013)) or two species (e.g., *Icerya purchasi*, *Palaeococcus fuscipennis*, *Drosicha corpulenta*, *Llaveia axin axin* (all Monophlebidae) (Szklarzewicz et al. 2006, 2013; Niżnik and Szklarzewicz 2007; Matsuura et al. 2009; Rosas-Pérez et al. 2014)), members of mealybug subfamily Pseudococcinae (von Dohlen et al. 2001; Thao et al. 2002; Kono et al. 2008; McCutcheon and von Dohlen 2011; Gatehouse et al. 2011; Husnik et al. 2013; Koga et al. 2013; Szabo et al. 2016), many armored scale insects) (Provencher et al. 2005)). In scale insects, the symbionts may be localized in bacteriocytes (in most scale insects) (e.g., Walczuch 1932; Buchner 1955, 1965; Tremblay 1990; von Dohlen et al. 2001; Szklarzewicz et al. 2006, 2013; Niżnik and Szklarzewicz 2007; Matsuura et al. 2009; Ramirez-Puebla et al. 2010; Gruwell et al. 2012) or may be dispersed in the cells of the fat body (in eriococcids *Acanthococcus aceris* and *Gossyparia spuria*) (Michalik et al. 2016). In the case of the presence of two or more types of symbionts, they are usually harbored in separate cells, e.g., in *Palaeococcus fuscipennis* and *Icerya purchasi*, one of the symbiont occupies the giant bacteriocytes, whereas the second one is localized in small epithelial cells surrounding each bacteriocyte (Szklarzewicz et al. 2006; Niżnik and Szklarzewicz 2007). A unique style of co-existence of two symbionts has been found in pseudocoocinae mealybugs, in which one type of symbiotic bacteria (gammaproteobacteria—closely related to the bacterium *Sodalis*) occurs inside the cells of other bacteria (betaproteobacteria *Tremblaya*) (von Dohlen et al. 2001; Thao et al. 2002; Kono et al. 2008; McCutcheon and von Dohlen 2011; Gatehouse et al. 2011; Husnik et al. 2013; Koga et al. 2013; Szabo et al. 2016). Results of recent molecular studies have demonstrated that both these bacteria, like the “co-primary symbionts” in Hemiptera: Auchenorrhyncha, are engaged in the synthesis of amino acids to the host insect (McCutcheon and von Dohlen 2011; Husnik and McCutcheon 2016).

The symbiotic systems of the members of the Putoidae family are not well known. Buchner (1955), using paraffin technique, described the distribution of symbionts and mode of their transmission between generations in *Puto antennatus* and *Puto superbus* (formerly treated as *Macrocerococcus superbus*, see “Introduction” for further details). Gruwell et al. (2014) identified the symbiotic bacteria harbored in five species of Putoidae from the Western Hemisphere: *Puto barberi*, *Puto echinocacti*, *Puto yuccae*, *Puto albicans*, and *Puto* sp. as gammaproteobacteria. Since the symbionts of *Puto barberi* appeared to be more phylogenetically distant from those in the remaining four species, the analysis of symbiotic microorganisms in the species *Puto superbus* from the Eastern Hemisphere may bring valuable arguments to the discussion concerning co-phylogeny of *Puto superbus* and its symbionts, as well as the phylogeny of the Putoidae family.

## Material and methods

### Insects

The adult females of *Puto superbus* (Leonardi 1907) were collected from the grass, *Arrhenatherum elatius* in Katowice (located in the south of Poland) and near Wolsztyn (in the west of Poland) in the months of June and July in the year 2012. Specimens destined for molecular analyses were collected in Katowice in June and July in the year 2014.

### Light and transmission electron microscopy analyses

The dissected ovaries and entire abdomens were fixed in 2.5% glutaraldehyde in 0.1 M phosphate buffer (pH 7.4), rinsed in the buffer with sucrose (5.8 g/100 ml), and postfixed in 1% buffered osmium tetroxidae (pH 7.4). The material was then dehydrated in a graded series of ethanol and acetone and embedded in epoxy resin Epon 812 (Serva, Germany). Semithin sections (1 μm thick) were stained with 1% methylene blue in 1% borax and examined and photographed with a Nikon Eclipse 80i light microscope. Ultrathin sections (90 nm thick) were contrasted with lead citrate and uranyl acetate and examined and photographed under a Jeol JEM 2100 electron microscope at 80 kV.

### DNA isolation, PCR, cloning, and sequencing

Total genomic DNA was isolated from adult females of *P. superbus* using a Sherlock AX DNA extraction kit (A&A Biotechnology), according to the manufacturer’s protocol. The DNA extracted was used for the molecular identification of symbionts colonizing the *P. superbus* tissue in the following experiments:

1. PCR reaction with universal, eubacterial primers: 10F (AGTTTGATCATGGCTCAGATTG) and 1507R (TACCTTGTTACGACTTCACCCCAG) (Sandström et al. 2001). The 16S rDNA of bacterial symbionts was amplified in 35 cycles and the following conditions: an initial denaturation step at 94 °C for 3 min, followed by 33 cycles at 94 °C for 30 s, 55 °C for 40 s, 70 °C for 1 min 40 s, and a final extension step of 5 min at 72 °C. Te PCR product was visualized on 1.5% agarose gel stained with Midori Green (Nippon) and purified using a GeneMATRIX PCR/DNA Clean-Up Purification Kit (Eurx);
2. Molecular cloning: the purified PCR product was cloned into pJET 1.2/blunt plasmid vector using a Clone JET PCR Cloning Kit (Thermo Scientific). The ligated mixtures were transformed into competent *Escherichia coli* TOP10F cells, which were prepared using an *E. coli* Transformer Kit (A&A Biotechnology). After 16 h, the occurrence of the bacterial 16S rDNA was confirmed by a diagnostic PCR reaction with the following primers: pJET For–GCCTGAACACCATATCCATCC and pJET Rev–GCAGCTGAGAATATTGTAGGAGAT.
3. Restrictive analysis: PCR products of 30 colonies were subjected to restriction analysis using an *MspI* restrictive enzyme. The plasmids from the selected colonies were isolated using a Plasmid Mini AX kit (A&A Biotechnology) and sequenced. The nucleotide sequences obtained were deposited into the GenBank database under the accession numbers KY558891-KY558893.

### Phylogenetic analysis

The phylogenetic analysis was performed based on sequences of 16S rDNA of *P. superbus* symbiont and selected gammaproteobacterial symbionts of hemipterans. The sequences homologous to the sequence obtained were found in the GenBank database using CLC MainWorkbench 7 software. The sequences were then edited using BioEdit Sequence Alignment Editor 5.0.9 (Hall 1999), and following this, the sequence alignments were generated using ClustalX 1.8 (Thompson et al. 1997). The base compositions of all the genes analyzed, as well as the genetic distances between the symbionts of scale insects belonging to the genus *Puto*, were estimated using MEGA 7 software (Kumar et al. 2016). The phylogenetic analysis was conducted using MrBayes 3.2.2 and MEGA 7 (Maximum likelihood) softwares (Ronquist and Huelsenbeck 2003). In the Bayesian analysis, four incrementally Metropolis coupling MCMC chains (3 heated and 1 cold) were run for ten million generations. The results of the Bayesian analysis were visualized using FigTree 1.4.0 software (Rambaut 2009).

## Results

### Molecular identification of symbionts of *Puto superbus*

**Fig. 1:**
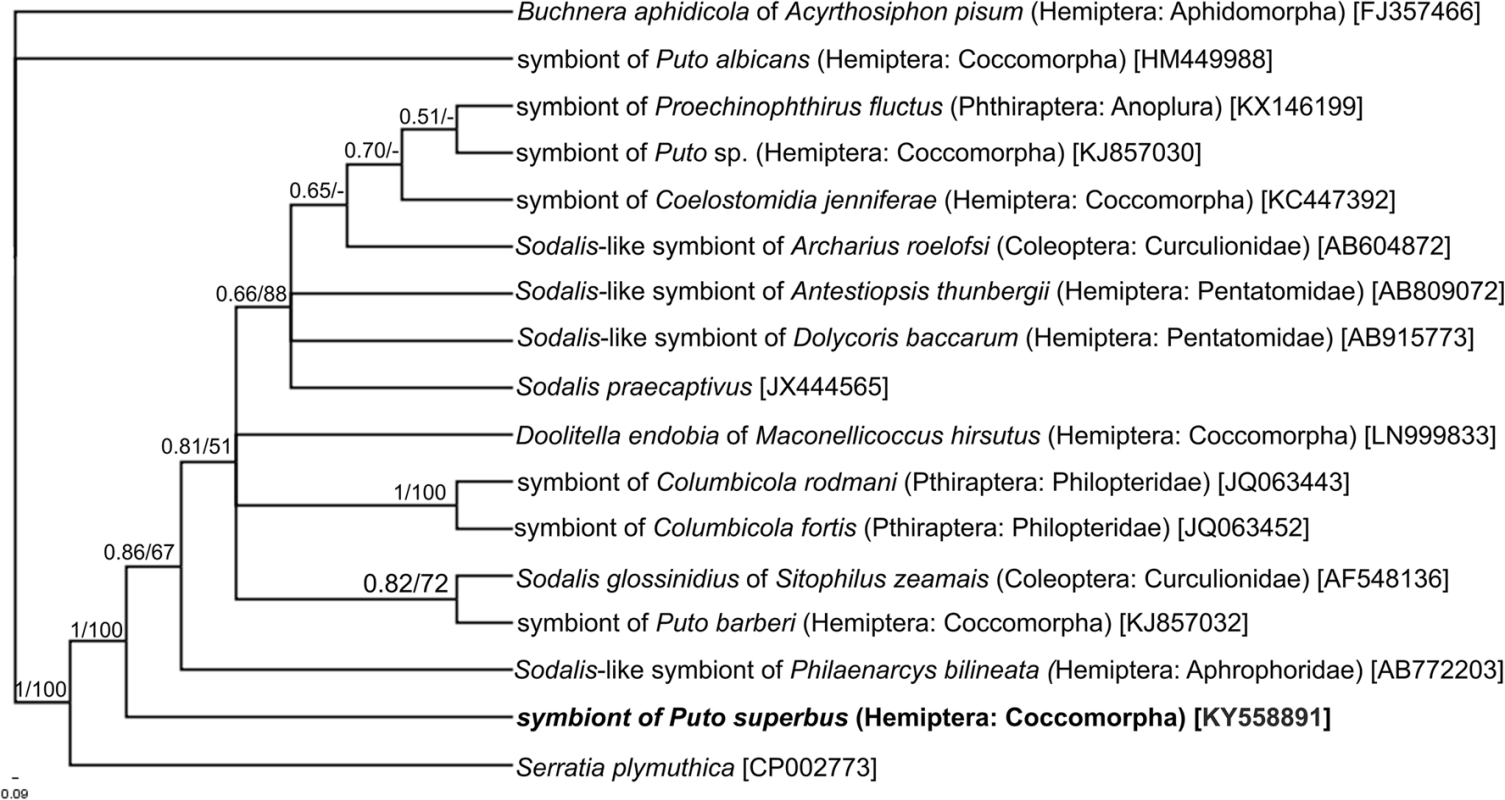
The cladogram showing the phylogenetic placement of the obligate symbiont of *Puto superbus*. The *numbers above the branches* indicate posterior and bootstrap values, respectively. The accession numbers of the sequences used in the phylogenetic analysis have been placed in *brackets*

The phylogenetic placement of symbionts occurring in the body of examined scale insect *Puto superbus* was determined based on their 16S rDNA sequences. Thirty positive colonies of bacteria *E. coli* obtained by molecular cloning were subjected to restrictive analysis with the *MspI* restrictive enzyme, and as a result, four different restrictive patterns were obtained. One of them, designated as no. 1, was clearly dominant whereas the other patterns (designated as no. 2, 3 and 4) were scarce. The selected clones (three of each type) were sequenced and the sequences obtained were compared with the data deposited in the GenBank using BLAST. The result of BLAST searches has indicated that the sequences of dominant restrictive patterns show 94–95% similarity to the bacteria belonging to the genus *Sodalis* (*S. glossinidius* and *S. praecoptirus*, respectively), whereas the sequences of pattern no. 2, 3, and 4 were identified as *Wolbachia* bacteria. All sequences represented by pattern no. 1 were identical; therefore, only one of them was used for the phylogenetic analysis. The 16S rDNA sequences of *Wolbachia* symbionts were slightly different from one another (with a 99% similarity). Since *Wolbachia* bacteria are widespread within different and unrelated insect groups, the sequence of 16SrDNA of *Wolbachia* was not used for phylogenetic analysis. The total length of sequences subjected to the phylogenetic analysis was 1322 bp, and the base composition was as follows: 21.7% T, 22.8% C, 26.3% A, and 29.1% G. The phylogenetic tree constructed using Bayesian analysis indicated that, based on 16S rDNA relationships, the obligate symbiont of *P. superbus* belongs to the Gammaproteobacteria within the Proteobacteria phylum and may be designated as a *Sodalis*-like symbiont (Fig. 1). Symbionts of scale insects belonging to *Puto* species (i.e., *Puto barberi*, *Puto albicans*, *Puto* sp., and examined *Puto superbus*), however, are placed in distinct lineages and are phylogenetically distant (Fig. 1). Sequence divergences between *Sodalis*-like symbionts of examined *P. superbus* and other scale insects belonging to genus *Puto* range from 2.7% (between *P. superbus* and *P. barberi*) to 14.7% (between *P. superbus* and *P. albicans*) (Table 1).

**Table 1:** Pairwise, genetic distances among 16S rDNA sequences of *Sodalis*-like symbionts of scale insects belonging to genus *Puto*

		1	2	3	4
1.	*Puto superbus*				
2.	*Puto albicans*	0.147			
3.	*Puto barberi*	0.027	0.147		
4.	*Puto* sp.	0.037	0.146	0.024	
5.	*Puto yuccae*	0.038	0.145	0.024	0.004

### Ultrastructure, distribution, and transovarial transmission of symbiotic microorganisms

**Fig. 2:**
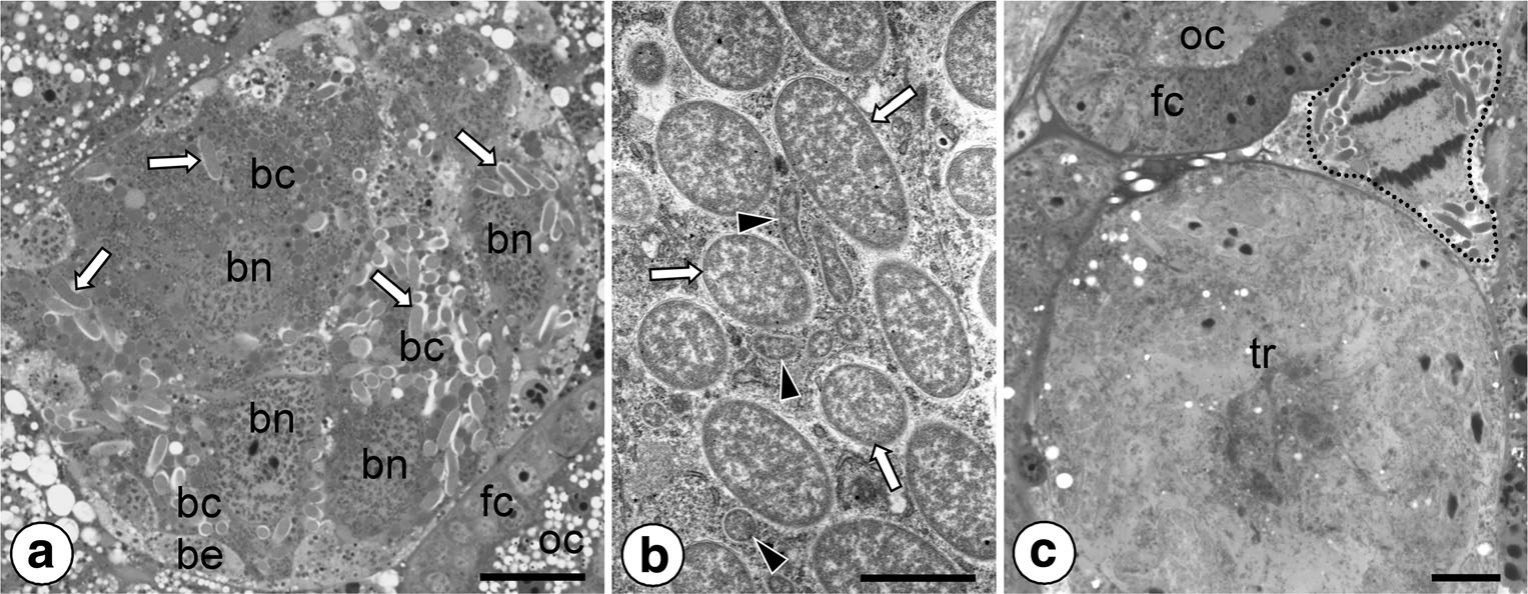
Distribution of symbiotic microorganisms in the body of *P. superbus.* **a** Fragment of the bacteriome (cross section). *White arrows* indicate large elongated *Sodalis*-allied symbionts. Bacteriocyte (*bc*), bacteriocyte nucleus (*bn*), bacteriome epithelium (*be*), follicular epithelium (*fc*), oocyte (*oc*). Methylene blue, *scale bar* = 20 μm. **b** Fragment of the bacteriocyte. Note large elongated *Sodalis-*like bacteria (*white arrows*) and small rod-shaped bacteria *Wolbachia pipientis* (*black arrowheads*). TEM, scale bar = 2 μm. **c** Bacteriocytes accompanying the ovarioles. Note division of the bacteriocyte (*encircled with black dotted line*). Follicular epithelium (*fc*), oocyte (*oc*), tropharium (*tr*). Methylene blue, *scale bar* = 20 μm

In adult females, elongated organs termed bacteriomes occur in the immediate vicinity of the ovaries (Fig. 2a). The bacteriomes are composed of numerous giant cells termed bacteriocytes and are surrounded by a thin layer of sheath cells (Fig. 2a). Ultrastructural observation did not reveal bacteria in the sheath cells. The bacteriocyte cytoplasm is filled with numerous elongated bacteria, and rare small rod-shaped bacteria, large nucleus, and ribosomes (Fig. 2a, b). The larger bacteria measure about 1.2–1.7 μm in diameter, whereas the smaller ones measure about 0.14–0.26 μm. The comparison of ultrastructural observations (i.e., shape and size of bacteria), along with the results of molecular analyses, has shown that larger bacteria represent *Sodalis*-allied symbionts, whereas these smaller ones correspond to the bacterium *Wolbachia pipientis*, which is widely distributed among invertebrates. It was observed that numerous separate bacteriocytes (i.e., not integrated into the bacteriome) are also scattered among ovarioles constituting the ovaries (Fig. 2c) (for further details concerning organization of ovary in *P. superbus*, see Michalik et al. 2013). Bacteriocytes accompanying ovarioles are less voluminous and are much more tightly packed with symbiotic bacteria (Fig. 2c) than bacteriocytes constituting the bacteriome. It was observed that the bacteriocytes accompanying the ovarioles undergo intense divisions (Fig. 2c).

**Fig. 3:**
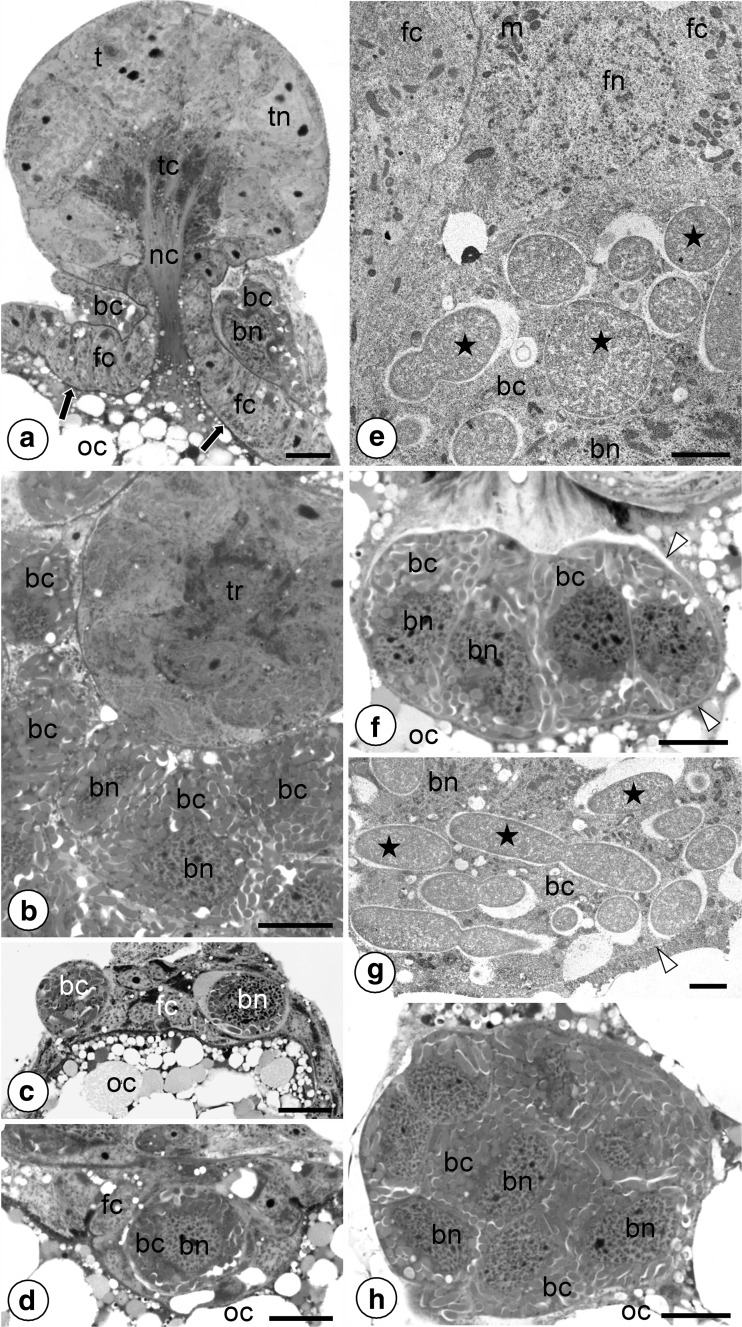
Consecutive stages of transovarial transmission of symbionts from one generation to the next. **a, b** The ovariole surrounded by bacteriocytes (*bc*) (**a** longitudial section, **b** cross section). Egg envelopes (*black arrows*), bacteriocyte nucleus (*bn*), follicular epithelium (*fc*), nutritive cord (*nc*), oocyte (*oc*), trophocyte (t), trophic core (tc), trophocyte nucleus (*tn*), tropharium (*tr*). Methylene blue, *scale bar* = 20 μm. **c–e** Bacteriocytes (*bc*) migrate through the follicular epithelium (*fc*) (longitudinal section). *Sodalis*-like bacteria (*black asterisks*), bacteriocyte nucleus (*bn*), follicular cell nucleus (*fn*), mitochondria (*m*), oocyte (*oc*). **c, d** Methylene blue, *scale bar* = 20 μm. **e** TEM, *scale bar* = 2 μm. **f, g** Bacteriocytes (bc) after passing through the follicular epithelium gradually gather in the invagination of the oolemma (longitudinal section). Bacteriocyte nucleus (*bn*), oocyte (*oc*), oolemma (*white arrowheads*), *Sodalis*-like bacteria (*black asterisks*). Methylene blue, *scale bar* = 20 μm. **h** A “symbiont ball” composed of numerous bacteriocytes (*bc*) (cross section). Bacteriocyte nucleus (*bn*), oocyte (*oc*). Methylene blue, *scale bar* = 20 μm

In females containing oocytes in the ovarioles in the stage of choriogenesis, the bacteriocytes begin to surround the neck region of ovarioles (i.e., the region between the tropharium and vitellarium) (Fig. 3a, b). At the time the bacteriocytes gather around the neck of the ovariole, the tropharium and the developing oocyte located in the vitellarium are connected by means of broad nutritive cord (Fig. 3a). The lateral and posterior aspects of the oocyte are surrounded by egg envelopes (Fig. 3a). The only region of the oocyte not covered with egg envelopes is the nutritive cord at which the tropharium joins the anterior pole of the oocyte (Fig. 3a). Next, the whole and intact bacteriocytes begin to invade the anterior region of the vitellarium (Fig. 3c). The bacteriocytes traverse the follicular epithelium migrating through the spaces between neighboring follicular cells (Fig. 3c–e). After passing through the follicular epithelium, the bacteriocytes gradually assemble in the perivitelline space (space between the oocyte and follicular epithelium) (Fig. 3f, g). Simultaneously, the tropharium and nutritive cord degenerate and the oocyte become surrounded by complete egg envelopes. In full-grown oocytes, the bacteriocytes become embedded in the deep invagination of oolemma in the form of a “symbiont ball” (Fig. 3h).

## Discussion

As stated in the “Introduction,” scale insects, unlike other hemipterans living in obligate symbiotic associations with microorganisms, are host to different species of symbionts. The results of the recent molecular analyses of symbionts hosted by different scale insect species (e.g., von Dohlen et al. 2001; Thao et al. 2002; Gruwell et al. 2007, 2010, 2014; Kono et al. 2008; Matsuura et al. 2009; Ramirez-Puebla et al. 2010; Gatehouse et al. 2011; Dhami et al. 2012; Rosenblueth et al. 2012; Koga et al. 2013; Rosas-Pérez et al. 2014; Michalik et al. 2016; Szabo et al. 2016) strongly support hypothesis of Koteja (1985) based on Buchner’s (1965) histological observations that such diverse symbioses are the result of the independent acquisition of free-living bacteria through the ancestors of extant coccoids. According to Koteja (1985), ancestral scale insects lived in forest litter, where they are permanently in contact with free-living bacteria. During further evolution, the diverged groups of scale insects changing the feeding behavior from saprophagic into plant sap-sucking have acquired bacteria which then co-evolved with them as their symbionts.

Molecular phylogenetic analyses have shown that the primary symbiont associated with *Puto superbus* is related to the gammaproteobacterium *Sodalis*, which is widely distributed among insects. The results of numerous recent papers have revealed that *Sodalis*-allied bacteria may fulfill the function of the primary symbiont (e.g., in the slender pigeon louse *Columbicola columbae*, louse fly *Craterina melbae*, weevil *Sitophilus oryzae*), secondary symbiont (e.g., in the tsetse fly *Glossina*, several species of psyllids, e.g., *Anomoneura mori*, *Trioza magnoliae*, *Cacopsylla myrthi*, in several species of aphids from the Lachninae family, e.g., *Cinara glabra*, *Eulachnus pallidnus*, *Eulachnus rileyi*, *Eulachnus mediterraneus*, *Nippolachnus piri*, stinkbugs, e.g., *Cantao ocellatus*, *Eucoryssus grandis*, *Antestiopsis thunbergi*) or may co-reside with other microorganisms as co-primary symbiont (e.g., in the leafhopper *Cicadella viridis*, in several species of spittlebugs, e.g., *Aphrophora quadrinotata*, *Philaenus soumarius*, *Lepyronia coleoptrata*, in pseudococcinae mealybugs) (Heddi et al. 1998; Dale and Maudlin 1999; Thao et al. 2000, 2002; Fukatsu et al. 2007; Nováková and Hypša 2007; Kono et al. 2008; Burke et al. 2009; Gruwell et al. 2010, 2014; Kaiwa et al. 2010, 2011; Gatehouse et al. 2011; Husnik et al. 2013; Husnik and McCutcheon 2016; Koga et al. 2013; Koga and Moran 2014; Matsuura et al. 2014; Michalik et al. 2014; Hosokawa et al. 2015; Manzano-Marin et al. 2017). *Sodalis*-like symbionts may be localized both intracellularly (e.g., in the cells of milk glands in the tsetse fly, in bacteriocytes in the leafhopper *Cicadella viridis*, psyllids and slender pigeon louse *Columbicola columbae*, inside the cells of other bacterium in mealybugs from the subfamily Pseudococcinae) as well as extracellularly (e.g., in the lumen of milk glands in the tsetse fly, in the gut appendages in stinkbugs). The situation in *Cicadella viridis* is of special interest because in this species, in contrast to the pseudococcinae mealybugs (in which *Sodalis*-like bacteria never occur individually—see “Introduction”), the *Sodalis*-allied symbionts both occur in their own bacteriocytes and they also co-inhabit bacteriocytes with the betaproteobacterium *Sulcia muelleri* (Michalik et al. 2014). The results of molecular phylogenetic analyses of *Sodalis*-like symbionts in members of the Putoidae family (Gruwell et al. 2014; this study—Fig. 1 and Table 1) have shown that these bacteria are phylogenetically distant. This fact supports the suppositions mentioned above and indicates that even in close relatives such as members of the same small family Putoidae, the *Sodalis* bacteria have been independently acquired several times.

The histological observations made by Walczuch (1932) and Buchner (1965), as well as results of more recent studies (von Dohlen et al. 2001; Szklarzewicz et al. 2006, 2013; Niżnik and Szklarzewicz 2007; Michalik et al. 2016), have shown that scale insects, in comparison with other insect taxa, exhibit an enormous diversity of the modes of symbiont inheritance. The symbiotic microorganisms associated with scale insects may infect the larval ovaries containing undifferentiated germ cells termed cystocytes (e.g., secondary symbiont in *Icerya purchasi* (Monophlebidae), primary symbiont in *Puto albicans* (Putoidae)) or the ovaries of adult females comprising vitellogenic or choriogenic oocytes (e.g., primary symbiont of *Icerya purchasi*, *Palaeococcus fuscipennis* (both Monophlebidae), *Acanthococcus aceris*, *Gossyparia spuria* (both Eriococcidae), and mealybugs (Pseudococcidae), the secondary symbiont of *Palaeococcus fuscipennis* (Monophlebidae)). In the case of infection of the older oocytes, the symbionts may infest the anterior pole of the oocyte (e.g., *Acanthococcus aceris*, *Gossyparia spuria*, mealybugs) or its posterior pole (e.g., *Icerya purchasi*, *Palaeococcus fuscipennis*) (Walczuch 1932; Buchner 1965; von Dohlen et al. 2001; Szklarzewicz et al. 2006, 2010, 2013; Niżnik and Szklarzewicz 2007; Michalik et al. 2016). It should be stressed that the mode of symbiont transmission relying on the infection of the anterior pole of the oocyte, as in *P. superbus*, is very rare among insects and has so far been observed in only scale insects. The manner of symbiont transmission observed in *P. superbus* is also unique in that the bacteriocytes which are whole and intact enter the ovarioles. So far, a similar phenomenon has been described in the *Puto* genus (Buchner 1955, 1965) and in whiteflies only (Buchner 1965; Costa et al. 1993; Szklarzewicz and Moskal 2001). It is worth adding that in cockroaches (Sacchi et al. 1988) and lice (Buchner 1965), the entire bacteriocytes penetrate the ovaries; however, the symbiotic bacteria are finally released from these cells and individually infest the ovarioles. According to Buchner (1965), the bacteriocytes in whiteflies degenerate during embryonic development, whereas in *Puto*, they enter the embryo. The transmission of symbionts in the *Puto* genus (*P. superbus* and *P. antennatus*) is of special interest in that their bacteriocytes differentiate into two types: (1) bacteriocytes which constitute the bacteriome and degenerate in the adult female (named by Buchner primary bacteriocytes) and (2) bacteriocytes accompanying the ovarioles which migrate to the ovarioles and are incorporated into the embryo during embryonic development (named by Buchner secondary bacteriocytes). Buchner (1955, 1965) observed that the differentiation of the bacteriocytes into two types takes place in the first larval instar. Both Buchner’s observations and our results have revealed that bacteriocytes associated with ovarioles divide intensively. It may be suggested that this phenomenon may result in a decrease of the volume of giant polyploid bacteriocytes. This, in turn, facilitates the migration of the bacteriocytes through the follicular epithelium.

Interestingly, the results of the preliminary studies of another member of the Putoidae family, *Puto albicans* (Szklarzewicz et al. 2010), indicate that its symbiotic bacteria are transmitted between generations in a completely different manner. Szklarzewicz et al. (2010) observed that, in this species, symbiotic bacteria already occur in cystocytes which build larval ovaries. This fact indicates that the primary symbionts of *P. albicans*, just as the secondary symbionts of *Icerya purchasi* (Niżnik and Szklarzewicz 2007), infect the dividing cystocytes. As a consequence, in both *P. albicans* and *I. purchasi*, after the differentiation of the cystocytes into oocytes and trophocytes, the symbionts occupy both these cell types. Since the microorganisms are transmitted to the progeny through only the oocytes, the bacteria residing in the trophocytes then migrate through the processes of trophocytes, trophic core, and nutritive cord into the developing oocyte. The different modes of symbiont transmission in species of the genus *Puto*, thus, support the hypothesis of the independent acquisition of symbionts in Putoidae.

It should be stressed that results of studies of the symbionts of the genus *Puto* (Szklarzewicz et al. 2010; Gruwell et al. 2014; this study), as well as results of previous studies on the organization of their ovaries (for further details, see Michalik et al. 2013), strongly support the current systematic position of these scale insects, i.e., in the separate Putoidae family within the archaeococcoid subgroup. This view is substantiated by the observation that the symbiotic bacterium *Tremblaya phenacola* in mealybugs from the family Pseudococcidae (subfamily Phenacoccinae), to which *P. superbus* was formerly classified (see “Introduction”), belongs to distinct group of bacteria, namely to the class Betaproteobacteria (Gruwell et al. 2010; Koga et al. 2013).

Both ultrastructural and molecular analyses have revealed that apart from the *Sodalis* bacteria, the *Wolbachia* bacteria are present in the bacteriocytes of all the specimens of *P. superbus. Wolbachia* bacterium, which is widely distributed among arthropods and nematodes, is regarded as playing variable roles for host insect. This means that the bacterium may feminize male embryos, kill male embryos, cause cytoplasmic incompatibility in infected males and uninfected females, and may also induce parthenogenesis (for further details, see Werren 1997; Stouthamer et al. 1999; Werren et al. 2008). The function of the bacterium in *P. superbus* remains unclear; however, both previous studies on ovaries (Michalik et al. 2013) as present studies on symbionts did not reveal the negative influence of these bacteria on the reproduction of the host insect. As we have analyzed only two populations of *P. superbus*, it still remains unknown whether this bacterium is present in specimens from other locations. In contrast to other insects, such as heteropterans (Kikuchi and Fukatsu 2003) and aphids (Augustinos et al. 2011), the prevalence of *Wolbachia* infections in scale insects has not been extensively examined to date. So far, apart from *P. superbus*, the bacterium *Wolbachia* has been found in a few species of coccoids representing three families: *Coelostomidia wairoensis* (Coelostomidiidae), *Icerya purchasi* and *Drosicha pinicola* (both Monophlebidae), and *Kerria lacca* (Kerridae) (Duron et al. 2008; Matsuura et al. 2009; Vashishtha et al. 2011; Dhami et al. 2012). Taking into account the fact that about 16% of insect species are infected by *Wolbachia* (Werren 1997), it seems reasonable to expect that in the near future, this bacterium will be detected in other species of scale insects.
